# Automatic query generation using word embeddings for retrieving passages describing experimental methods

**DOI:** 10.1093/database/baw166

**Published:** 2017-01-10

**Authors:** Ferhat Aydın, Zehra Melce Hüsünbeyi, Arzucan Özgür

**Affiliations:** Department of Computer Engineering, Boğaziçi University, TR-34342 Bebek, Istanbul, Turkey

## Abstract

Information regarding the physical interactions among proteins is crucial, since protein–protein interactions (PPIs) are central for many biological processes. The experimental techniques used to verify PPIs are vital for characterizing and assessing the reliability of the identified PPIs. A lot of information about PPIs and the experimental methods are only available in the text of the scientific publications that report them. In this study, we approach the problem of identifying passages with experimental methods for physical interactions between proteins as an information retrieval search task. The baseline system is based on query matching, where the queries are generated by utilizing the names (including synonyms) of the experimental methods in the Proteomics Standard Initiative–Molecular Interactions (PSI-MI) ontology. We propose two methods, where the baseline queries are expanded by including additional relevant terms. The first method is a supervised approach, where the most salient terms for each experimental method are obtained by using the term frequency–relevance frequency (tf.rf) metric over 13 articles from our manually annotated data set of 30 full text articles, which is made publicly available. On the other hand, the second method is an unsupervised approach, where the queries for each experimental method are expanded by using the word embeddings of the names of the experimental methods in the PSI-MI ontology. The word embeddings are obtained by utilizing a large unlabeled full text corpus. The proposed methods are evaluated on the test set consisting of 17 articles. Both methods obtain higher recall scores compared with the baseline, with a loss in precision. Besides higher recall, the word embeddings based approach achieves higher *F*-measure than the baseline and the tf.rf based methods. We also show that incorporating gene name and interaction keyword identification leads to improved precision and *F*-measure scores for all three evaluated methods. The tf.rf based approach was developed as part of our participation in the Collaborative Biocurator Assistant Task of the BioCreative V challenge assessment, whereas the word embeddings based approach is a novel contribution of this article.

Database URL: https://github.com/ferhtaydn/biocemid/

## Introduction

The functions of proteins are often modulated through their interactions with other proteins. Protein–protein interactions (PPIs) play important roles in many biological processes including cell cycle control, DNA replication, translation, transcription and metabolic and signaling pathway (1). A number of databases such as BioGrid (2), IntAct (3), DIP (4), MINT (5) and BIND (6) have been developed to store PPI information in well structured format in order to facilitate data retrieval and systematic analysis. The PPI information in these databases is extracted manually by human curators from the published literature. However, manual curation is a laborious and time consuming task. Therefore, it is only able to handle a small fraction of the rapidly growing biomedical literature (7). In order to address this challenge, several text-mining studies have been conducted for automatically extracting information from the published articles. The community-wide shared tasks such as BioCreative (8–10) and BioNLP (11–13) have played important roles for promoting research in this area. Being one of the main tasks in these community-wide efforts, extracting interactions among proteins has gained significant attention from the researchers. Although improvements have been obtained in extracting PPIs from text in the recent years (14, 15), enriching PPIs with context information including the experimental methods used to detect the PPIs has not been well studied yet (16). Various experimental methods such as ‘affinity capture’, ‘two-hybrid’ and ‘coimmunoprecipitation’ are available for detecting protein interactions (1). Experimental methods have different degrees of resolution, confidence and reliability. Therefore, besides the existence of an interaction between a pair of proteins, the experimental conditions in which this interaction was observed are also very important for the interpretation and assessment of the interaction (16).

The problem of identifying the experimental methods used to detect a given PPI in an article was tackled by the Interaction Method Subtask (IMS) of the BioCreative II challenge (14). Two teams participated in the sub-task (17, 18). Rinaldi *et al.* (17) obtained promising results by using mostly manually crafted patterns for matching the experimental method terms in the provided ontology against the full text article including the PPI. Ehrler *et al.* (18) used a pattern matching and vector space retrieval based model. A similar task, namely the Interaction Method Task (IMT), was also addressed at the BioCreative III challenge (8, 19). The goal in the IMT task at BioCreative III was to identify the experimental methods in a given full text article and map them to the interaction detection method terms in the PSI-MI ontology (20, 21). The positions of the experimental methods in the articles were not required to be identified.

Most previous studies on experimental method detection, including the ones in the BioCreative challenges, used pattern matching and/or machine learning based approaches. In the pattern matching based approach the experimental method names in text are matched against the terms in a lexicon or ontology such as the PSI-MI ontology using usually hand-crafted patterns (22–25). Pattern matching based methods are able to identify the positions of the experimental method mentions in the articles. However, they fail to identify the experimental methods when they occur in forms that do not match the designed patterns. In order to handle approximate matches, Matos et al. developed an Information Retrieval based system, where the test documents are indexed and searched for experimental methods using the Lucene search library (26). In the machine learning based approach, the task of experimental method detection is in general formulated as a text classification task, where the classes are defined as the experimental methods and the goal is to classify the articles into zero or more of these classes. Machine learning based methods obtained promising results in the BioCreative III challenge, where different classification algorithms such as Naive Bayes (27), Random Forest (28), Support Vector Machines (29), and Logistic Regression (29) were utilized. Machine learning based methods classify articles as containing a certain experimental method or not. Experimental methods can be detected, even if they don’t occur with their standard names or synonyms. However, the positions of the experimental methods in the articles are not identified.

**Figure 1.**
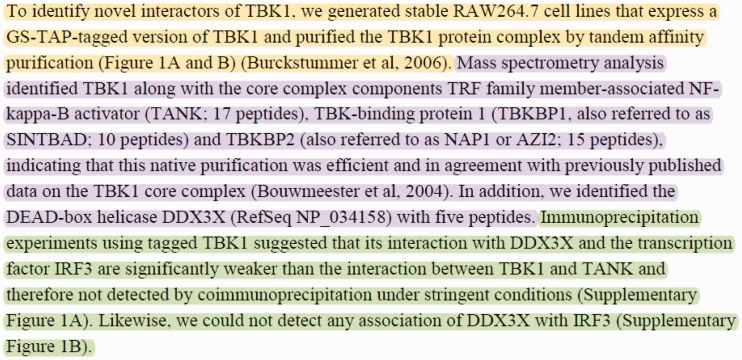
Sample text with multiple PPIs and experimental methods taken from the Results section of (30). The text describes three experimental interaction detection methods used to identify the proteins interacting with the ‘TBK1’ protein. The passages describing the experimental interaction detection methods ‘tandem affinity purification’ (MI:0676), ‘mass spectrometry studies of complexes’ (MI:0069), and ‘coimmunoprecipitation’ (MI:0019) are highlighted with yellow, purple and green, respectively.

In this article, we approach the problem of experimental method detection as a passage retrieval task. We target identifying passages (i.e. sequences of sentences) where certain experimental methods are described. In many cases, experimental method descriptions span multiple sentences. Passage-level retrieval is especially crucial for articles in which multiple PPIs and experimental methods are mentioned. Passage-level retrieval can help mapping PPIs to their corresponding experimental methods. For instance, consider the sample text from (30) shown in Figure 1. The text describes three experimental methods used to identify the proteins interacting with the ‘TANK-binding kinase 1’ (TBK1) protein. The passages describing the experimental methods ‘tandem affinity purification’ (MI:0676), ‘mass spectrometry studies of complexes’ (MI:0069) and ‘coimmunoprecipitation’ (MI:0019) are highlighted with yellow, purple and green, respectively. Different PPIs were observed by using these three experimental methods. For example, the passage about the ‘coimmunoprecipitation’ experiment (shown in green) states that no interactions were observed between the protein pairs TBK1-DDX3X, TBK1-IRF3 and DDX3X-IRF3 by using the ‘coimm unoprecipitation’ experimental technique. This example illustrates that experimental method descriptions may span multiple sentences. In addition, it demonstrates that identifying the passages describing the experimental methods is important for resolving which method detected which of the PPIs described in text.

We describe two query matching approaches for retrieving passages related to physical PPI detection methods from articles. The first approach is based on generating queries using the term frequency–relevance frequency (tf.rf) metric and was developed as part of our participation in the BioCreative V BioC Track (31). The aim of the BioC track was to develop BioC-compatible (32) modules integrated together to form a text-mining system to assist biocurators (33, 34). Our second approach is based on generating queries by using the word embeddings of the experimental method names (i.e. the canonical name and synonyms) in the PSI-MI ontology (The ontology is available at http://www.ebi.ac.uk/ols/beta/ontologies/mi) (PSI-MI, Version: 2.5, RRID:SCR_010710). We obtained the word embeddings by using the ‘word2vec’ (Word2vec Tool: http://word2vec.googlecode.com/; Revision-42:http: //word2vec.googlecode.com/svn/trunk/) tool (word2 vec, Version: Revision 42, RRID:SCR_014776) (35), which is an efficient implementation of neural networks based learning techniques for constructing word vectors from large unlabeled data sets with billions of words (36). As an additional contribution of this study, a data set consisting of 30 full text articles is manually annotated for passages describing experimental methods and made publicly available.

## Materials and methods

### Data set

To the best of our knowledge, there does not exist a data set annotated for experimental interaction detection methods (with MI ontology identifiers) at the passage level (with exact location in the article). The available data sets for experimental methods are annotated at the article level (e.g. the BioCreative II IMS and BioCreative III IMT data sets (BioCreative, RRID:SCR_006311) (14, 19)). In other words, only the list of experimental methods for each article is provided. Therefore, we manually annotated a data set of full text articles at the passage level by selecting a subset of the BioCreative III IMT task data set. The subset of articles was selected according to the availability of the articles in ‘PMC Open Access’ (http://www.ncbi.nlm.nih.gov/pmc/) (PubMed Central, RRID:SCR_004166) (37), as full text, as well as their availability in BioC format. 30 articles from this subset were randomly selected and annotated for passages (i.e. sequences of sentences) that describe an experimental method as an evidence for a physical PPI and for the specific method that each passage describes by two annotators who have natural language processing and information retrieval background. The disagreements between the two annotators were resolved collaboratively. Then, the annotations of the test set consisting of 17 articles were checked, validated, and corrected whenever necessary by a domain expert. These final annotations were used as the gold standard. The Inter Annotator Agreement (IAA) over the test set is computed by comparing the combined annotations of the two annotators (after resolving the disagreement between them) against the gold standard test set checked by the domain expert. The evaluation approach described in ‘Evaluation’ section is used to measure IAA precision, recall and *F*-measure (38), which are computed as 0.787, 0.937 and 0.856, respectively. The annotated data set is publicly available (https://github.com/ferhtaydn/biocemid/tree/odj/files/published_dataset) (Biocemid, RRID:SCR_014779).

The data set of 30 articles is split into two parts, where the first part comprises 13 articles and is used as training set in the tf.rf based approach and as validation set in the word embeddings based approach. The remaining 17 articles, which were checked and validated by the domain expert, are used as test set for all methods developed in this study. The total number of annotated passages, the total number of paragraphs which have at least one annotated passage, and the total number of paragraphs which do not contain any annotated passages in the data set of 30 articles are 370, 292 and 1194, respectively.

**Figure 2.**
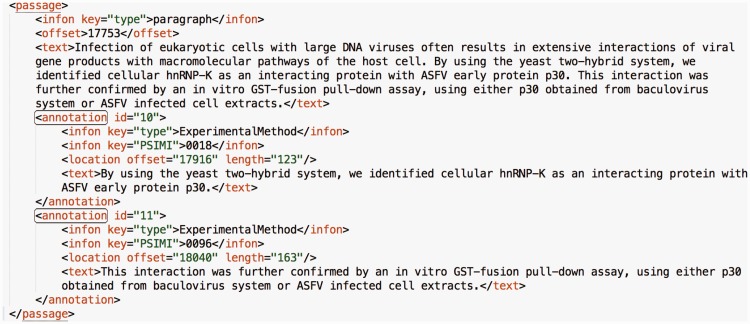
A sample annotation from a paragraph of an article in the data set. Each annotation has an identifier that is incremented by one throughout the article. Moreover, the value of the ‘type’ infon is static and set to ‘ExperimentalMethod’ for all annotations. The value of the ‘PSIMI’ infon is set to the PSI-MI identifier of the interaction detection method. The ‘text’ tag holds the annotated sentence(s). The ‘location’ tag holds the position of the annotated portion in the article with the ‘offset’ and ‘length’ attributes.

A sample annotation from a paragraph of an article in the data set is shown in Figure 2. Each annotation has an identifier that is incremented by one throughout the article and two infons, which store key-value pairs with any required information in the context (32). The value of the ‘type’ infon is set to ‘ExperimentalMethod’ for all annotations and the value of the ‘PSIMI’ infon is set to the PSI-MI identifier of the interaction detection method. The ‘text’ tag holds the annotated sentence(s). The ‘location’ tag holds the position of the annotated portion in the article with the ‘offset’ and ‘length’ attributes. As illustrated in Figure 2, different passages (sequences of sentences) in a paragraph can be annotated with different experimental methods. It is also possible that multiple experimental methods are explained in the same passage of a paragraph. In this case, the corresponding passage of the paragraph is annotated with each experimental method separately. If a paragraph comprises a continuous and coherent explanation of one experimental method, then the whole paragraph is annotated with that method only.

The articles are annotated by considering 103 interaction detection methods (https://github.com/ferhtaydn/biocemid/blob/odj/files/103_methods.txt) in the PSI-MI ontology (the nodes under ‘MI:0045’ (http://purl.obolibrary.org/obo/MI_0045) which defines ‘experimental interaction detection’). The annotation statistics for the 35 interaction detection methods that are annotated in at least one article in the data set are shown in Table 1. The PSI-MI identifiers of the methods, their canonical names in the PSI-MI ontology, the number of articles each method occurs in, as well as the total number of passages annotated for each method are presented in the table. Fifteen interaction detection methods are annotated in only one article and seven methods are annotated in only one passage. The most common methods at the article-level (i.e. annotated in the highest number of different articles) are ‘pull down’, ‘coimmunoprecipitation’, ‘two hybrid’, ‘anti bait coimmuno precipitation’ and ‘anti tag coimmuno precipitation’. The most common methods at the passage-level are ‘two hybrid’, ‘coimmunoprecipitation’, ‘pull down’, ‘nuclear magnetic resonance’, ‘chromatin immunoprecipitation assay’, ‘anti bait coimmunoprecipitation’ and ‘x-ray crystallography’.

**Table 1.** List of experimental interaction detection methods which are annotated in at least one article in the manually annotated data set

Id	Name	Articles	Passages
MI:0004	affinity chromatography technology	3	5
MI:0006	anti bait coimmunoprecipitation	8	23
MI:0007	anti tag coimmunoprecipitation	7	14
MI:0014	adenylate cyclase complementation	2	2
MI:0017	classical fluorescence spectroscopy	1	4
MI:0018	two hybrid	10	54
MI:0019	coimmunoprecipitation	14	49
MI:0029	cosedimentation through density gradient	1	1
MI:0030	cross-linking study	2	5
MI:0040	electron microscopy	1	4
MI:0053	fluorescence polarization spectroscopy	1	1
MI:0054	fluorescence-activated cell sorting	3	4
MI:0055	fluorescent resonance energy transfer	3	10
MI:0065	isothermal titration calorimetry	3	8
MI:0071	molecular sieving	4	9
MI:0077	nuclear magnetic resonance	4	27
MI:0081	peptide array	1	4
MI:0096	pull down	15	43
MI:0104	static light scattering	1	1
MI:0107	surface plasmon resonance	2	3
MI:0114	x-ray crystallography	5	21
MI:0276	blue native page	1	2
MI:0402	chromatin immunoprecipitation assay	5	24
MI:0411	enzyme linked immunosorbent assay	2	3
MI:0412	electrophoretic mobility supershift assay	1	2
MI:0413	electrophoretic mobility shift assay	1	6
MI:0416	fluorescence microscopy	5	15
MI:0419	gtpase assay	2	4
MI:0423	in-gel kinase assay	1	1
MI:0426	light microscopy	1	1
MI:0663	confocal microscopy	3	6
MI:0676	tandem affinity purification	1	4
MI:0809	bimolecular fluorescence complementation	1	8
MI:0858	immunodepleted coimmunoprecipitation	1	1
MI:0889	acetylase assay	1	1

### Methodology

**Figure 3.**
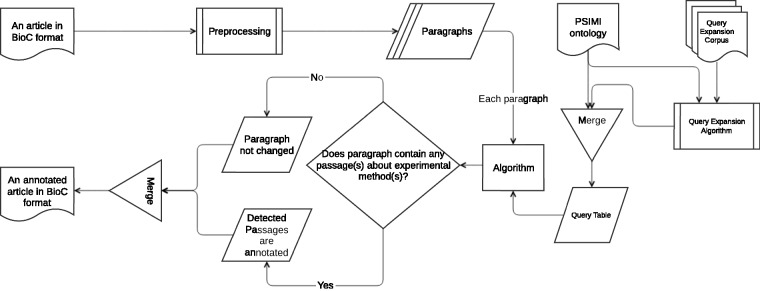
Overall system workflow.

An information retrieval based system for identifying passages that describe an experimental method as evidence for physical PPI is developed (Biocemid, RRID:SCR_014779). The overall workflow of the system is shown in Figure 3. The system pipeline takes a BioC article as input, processes it, and returns the article with the annotated passages for experimental methods in BioC format as output. ‘The BioC Java library’ (https://sourceforge.net/projects/bioc/files/BioC_Java_1.0.1.tar.gz/download) (BioC Java library, Version: 1.0.1, RRID:SCR_014777) (32) is used to read, modify, and re-create the BioC files.

In the preprocessing step a rule-based sentence splitting method, which we developed based on the period followed by a space pattern, is used. The infon types such as ‘title’, ‘table caption’, ‘table’, ‘ref’, ‘footnote’ and ‘front’ are excluded, since the text of some of these infon types are not sentences, but may contain experimental method relevant keywords. In order to reduce the number of false positives (FPs), paragraphs tagged with these infon types are not used for query matching. Moreover, even if paragraphs are tagged with the infon types that we do not exclude, they are not used for query matching if they comprise less than five words. We observe that such short paragraphs with infon types that we do not exclude, in general result due to incorrect tag assignment during BioC format conversion. For example, a header may be tagged with ‘paragraph’ infon instead of ‘title’, and the text may be ‘Pull-down Experiment Results’. The ‘Stanford CoreNLP toolkit’ (http://stanfordnlp.github.io/CoreNLP/index.html and http://nlp.stanford.edu/software/stanford-corenlp-full-2015-12-09.zip) (Stanford CoreNLP, Version: 3.6.0, RRID:SCR_014778) (39) is used to tokenize the sentences. At the tokenization phase, punctuation marks, braces, left and right parentheses, brackets, digits, floats etc. are removed from the sentences.

Three query matching based algorithms are designed to retrieve passages that describe specific experimental methods. All three algorithms share the same main idea that a query is generated for each experimental method and included in the query table. The queries in the query table are used to match against the paragraphs in the input article to annotate the passages with experimental methods. The article is returned from the pipeline either unchanged or annotated for the passages with the matching experimental methods.

Each algorithm is described in detail in the following sections.

***Baseline for query matching.*** The baseline algorithm defines an initial query for each experimental method by using the names of the experimental method in the PSI-MI ontology. For example, the initial queries for the ‘affinity chromatography technology’, ‘two hybrid’ and ‘pull down’ experimental methods are shown in Table 2. Although ‘pull down’ only has its name without any synonyms in the ontology, ‘affinity chromatography technology’ and ‘two hybrid’ have more than one synonyms. The algorithm uses the terms in the initial queries of experimental methods to detect relevant passages. Although determining the minimum performance line to be improved, the baseline algorithm is also designed to provide a base for the construction of the other two algorithms by expanding the initial queries.

**Table 2.** The initial queries for the ‘affinity chromatography technology’ (MI:0004), ‘two hybrid’ (MI:0018) and ‘pull down’ (MI:0096) experimental methods

MI:0004	MI:0018	MI:0096
affinity chromatography technology	two hybrid	pull down
affinity chrom	two-hybrid	
affinity purification	yeast two hybrid	
	2 hybrid	
	2-hybrid	
	y2h	
	classical two hybrid	
	gal4 transcription regeneration	
	2h	

The sentences in the paragraphs are matched against the query table of the initial queries for each experimental method. The initial queries contain terms, which can be word unigrams, bigrams or trigrams. If a sentence contains a term from the initial query of an experimental method, the sentence is annotated with that experimental method. If there are successive sentences with the same annotation, they are concatenated under one annotation tag. As a result, sentences or groups of sentences (passages) in paragraphs are annotated for experimental methods.

Two algorithms are developed on top of the baseline for expanding the initial query. The first algorithm is a supervised approach and uses a training set of articles annotated for passages with experimental methods. The most salient query terms are selected based on the tf.rf term weighting metric (40). The second algorithm is an unsupervised approach and utilizes a large unlabeled corpus for query expansion based on the word embeddings of the initial query terms.

***tf.rf-based query generation.*** The texts under the ‘annotation’ tags in the paragraphs (see Figure 2) of the manually annotated BioC articles in our data set were used as input for the tf.rf method. These texts were filtered according to each experimental method, split into sentences, and tokenized. The frequency of each token was calculated and token-frequency tuples were prepared. These tuples were used to calculate the weight of each token with the tf.rf method as follows.

(1) $$\text{tf}.\text{rf}=\text{tf}*\;{\text{log}}_{2}(2+\frac{a}{\text{max}(1,c)})$$

tf is the number of times the token occurs in the passages annotated for the given experimental method (i.e. passages in the positive category), *a* is the number of passages in the positive category that contain the token, and *c* is the number of passages in the negative category (i.e. passages annotated with other experimental methods) that contain the token. The intuition behind rf is that a term that occurs more in the positive category compared with the negative category has more discriminating power.

For each experimental method the terms are ranked by their tf.rf weights and manually examined to create the first tier tf.rf and second tier tf.rf term lists and the initial query of that experimental method is expanded by these lists. The first tier tf.rf list consists of high scored relevant tf.rf terms, whereas the second tier tf.rf list consists of lower scored, yet still relevant terms. An example expanded query for the ‘pull down’ experimental method is shown in Table 3. We also investigated selecting the first and second tier term lists automatically. Table 4 shows the expanded query for the ‘pull down’ experimental method generated by selecting the top seven terms based on their tf.rf scores as first tier terms, and the next top seven terms as second tier terms. Similarly, Table 5 shows the expanded query when the top 10 terms based on their tf.rf scores are selected as first tier terms and the next top 10 terms are selected as second tier terms. The names of the experimental methods are excluded from the first and second tier lists even if they have high tf.rf weights, since they are already included in the initial query.

**Table 3.** Expanded query for the ‘pull down’ experimental method

Names	Tier 1 Terms	Tier 2 Terms
pull down	pull-down	flag-tagged
	down	pull
	pulled	pulled-down
	gst	gst-fusion
	his-tagged	glutathione
	s-transferase	glutathione-sepharose
	affinity	

The names are extracted from the PSI-MI ontology. The Tier 1 and Tier 2 terms are extracted manually based on tf.rf weights.

**Table 4.** Expanded query for the ‘pull down’ experimental method

Names	Tier 1 Terms	Tier 2 Terms
pull down	pull-down	binding
	gst	gst-hnrnp-k
	rab5	recombinant
	appl1	interaction
	down	his-tagged
	proteins	protein
	melk	mutations

The names are extracted from the PSI-MI ontology. The Tier 1 and Tier 2 terms are constructed automatically from the first 7 and second 7 top terms of tf.rf weights.

**Table 5.** Expanded query for the ‘pull down’ experimental method

Names	Tier 1 Terms	Tier 2 Terms
pull down	pull-down	interaction
	gst	his-tagged
	rab5	protein
	appl1	mutations
	down	pull
	proteins	used
	melk	gtp
	binding	assay
	gst-hnrnp-k	fusion
	recombinant	figure

The names are extracted from the PSI-MI ontology. The Tier 1 and Tier 2 terms are constructed automatically from the first 10 and second 10 top terms of tf.rf weights.

The sentences in the paragraphs are matched against the created queries for each experimental method. First, the names list of the expanded query is used. The names list contains terms which can be unigrams, bigram, or trigram (word-level). On the other hand, the first and second tier lists only consist of unigrams. The names found in the sentence have weight of 1.0. The terms in the first and second tier lists are searched in the sentences. A matching term from the first tier list is assigned the weight of 0.50, whereas a matching term from the second tier list is assigned the weight of 0.25. These weights have been set without tuning, but heuristically by giving full weight to a name/synonym in the PSI-MI ontology, half of this weight to a term from the first tier list, and 25% of this weight to a term from the second tier list. The threshold for selecting a sentence as relevant to an experimental method is set as 1.0. That means, existence of a name or a synonym of an experimental method in the sentence is enough to annotate the sentence with the corresponding experimental method, but if there is no name or synonym in the sentence, at least one Tier 1 term and two Tier 2 terms, or two Tier 1 terms, or four Tier 2 terms are need for annotation. The previous and next sentences of the selected sentence are also processed to check whether they are relevant to the same experimental method or not. If the previous and next sentences of the annotated sentence obtain the highest score for the same experimental method and if this score is ≥0.50 (i.e. contains at least one Tier 1 term or two Tier 2 terms), they are annotated with the same experimental method. All the successive sentences with the same annotation are concatenated under one annotation tag. As a result, sentences or groups of sentences (passages) in paragraphs are annotated for experimental methods.

***Word embeddings based query generation.*** In distributional models, the distributed representations of words are modeled by assuming that word similarity is based on the similarity of observed contexts. In other words, if two words tend to occur in similar contexts, it is likely that they also have similar semantic meanings. The distributed representations of words are generally implemented in continuous vector space models (i.e. word embeddings), where each word is represented as a point in the vector space. The coordinates of the words are determined according to the context items around them. Therefore, similar words are mapped to nearby points (41).

‘Word2vec’ (word2vec, RRID:SCR_014776) is an efficient implementation for unsupervised learning of word embeddings from an unlabeled corpus. It provides two predictive models; the Continuous Bag-of-Words model (CBOW) and the Skip-Gram model. The CBOW model predicts target words from source context words, while the Skip-Gram model does the inverse and predicts source context-words from the target words (35).

In this study, we used ‘word2vec’ to expand the initial experimental method queries consisting of the PSI-MI ontology terms by using word embeddings learned from a large unlabeled biomedical corpus. A set of 691,558 full text articles from the ‘PMC Open Access’ (http://www.ncbi.nlm.nih.gov/pmc/tools/openftlist/) database (PubMed Central, RRID:SCR_004166) is used as unlabeled data. All the articles are passed from a preprocessing pipeline. The Stanford CoreNLP tool is used for conversion of the data to lower case, tokenization and sentence splitting. Then, the punctuation marks are removed using a manually prepared list of punctuation marks. The numeric and non-ascii characters are also removed (Data Cleaning Code of this Study https://github.com/ferhtaydn/stopword_remover /tree/odj). After the preprocessing steps, all 691,558 articles are merged into a single text file in order to use as input for ‘word2vec’.

Since experimental method names generally consist of multiple words, vectors for words as well as phrases, which consist of up to four words (bigram, trigrams and fourgrams), are required. The phrases are obtained by running ‘word2phrase’ (http://word2vec.googlecode.com/svn/trunk/word2phrase.c), (word2vec, Version: Revision 42, RRID: SCR_014776), which uses bigram statistics to form phrases, twice (i.e. consecutively) with the default parameters on the preprocessed unlabeled data. The minimum word occurrence count is set as 5, the threshold parameter is set as 200 and 100 for the first and second runs, respectively. The phrases are treated as individual tokens like words (during training). The resulting data set contains 2,241,223,681 total and 3,229,270 unique tokens. This data set is given as unlabeled input data to the ‘word2vec’ tool, which is run with the ‘Hierarchical Softmax’ (42, 43) based training algorithm and the ‘Skip-Gram’ (35) architecture with the suggested default settings for the parameters. Context window size, sub-sampling rate, and training iteration count are set as 10, 1e−4 and 15, respectively. Minimum word occurrence count is set as 10. In other words, words appearing <10 times are removed. As a result, word vectors with size 200 are generated.

The word vectors of an experimental method’s names (and synonyms) in the PSI-MI ontology are used to expand the initial query for the experimental method. The word vectors for some terms of the initial queries could not be constructed by ‘word2vec’ because of the insufficient data related to those terms in the input unlabeled data set. For example, for the initial query of the ‘two hybrid’ (MI:0018) experimental method consisting of the terms ‘two hybrid’, ‘two-hybrid’, ‘yeast two hybrid’, ‘2 hybrid’, ‘2-hybrid’, ‘y2h’, ‘classical two hybrid’, ‘gal4 transcription regeneration’ and ‘2h’, only the vectors of the ‘2-hybrid’, ‘two-hybrid’, ‘y2h’ and ‘2h’ terms were constructed. For each term (which has a word vector) in the initial query, the top 100 terms whose word vectors are most similar (in terms of cosine similarity) to the word vector of the initial query term are retrieved by using the modified version of the ‘distance’ (https://github.com/ferhtaydn/word2vec_extension/blob/odj/distance_files.c) component of ‘word2vec’. Then, for each term in the initial queries, the top 100 similar terms are manually analyzed. It is observed that for ambiguous initial query terms such as the ‘2h’ initial query term of the ‘two hybrid’ experimental method, non-relevant terms with high cosine similarity scores are retrieved. Therefore, such ambiguous terms are removed from the initial queries of the corresponding experimental methods. The list of terms that are removed are (MI:0016, cd), (MI:0018, 2h), (MI:0053, fps), (MI:0055, ret), (MI: 00 99 , spa), (MI:0104, sly), (MI:0112, myth), (MI:0 114, x-ray), (MI:0226, ice), (MI:0419, gtpase), (MI:0428, microscopy), (MI:0437, trihybrid), (MI:0676, tap), (MI: 0728, kiss) and (MI:0825, x-ray).

**Figure 4.**
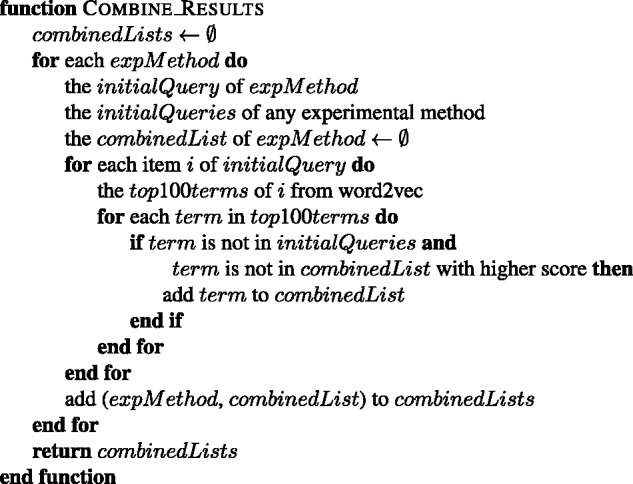
The algorithm for combining the word2vec results of each experimental method into one list.

**Figure 5.**
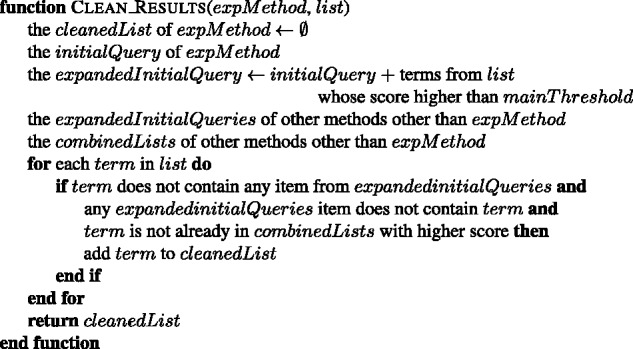
The algorithm for cleaning the given list of an experimental method.

**Figure 6.**
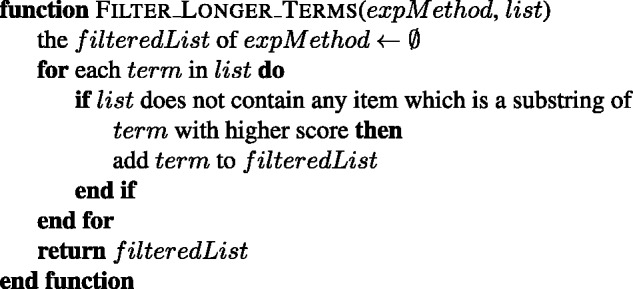
The algorithm for filtering the longer terms with lower scores from the given list of an experimental method.

**Figure 7.**
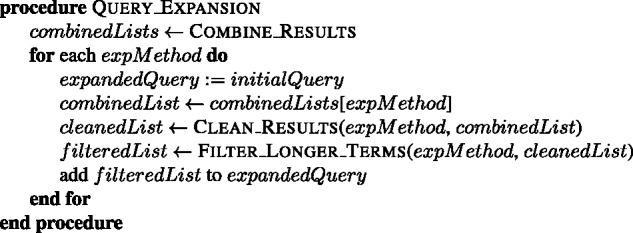
The query expansion algorithm of wor2vec approach.

For each term in the initial query of an experimental method, the most similar 100 terms are obtained. If the initial query contains *k* terms, then a pool of k*100 terms is obtained for the corresponding experimental method. A list of terms for each experimental method is created from its pool of terms by removing: (i) the duplicate terms by leaving the term with the highest cosine similarity score; (ii) the terms that are already listed as a name or synonym of an experimental method in the PSI-MI ontology (see the combine function in Figure 4); (iii) the terms which contain a name from an initial query of another experimental method, e.g. ‘gst-pull-down-assay’ and ‘pull’ are removed from the ‘two hybrid’ results; (iv) the terms which have a higher cosine score in the list of another experimental method, e.g. ‘coprecipitated, 0.82’ is removed from the results of ‘coimmunoprecipitation’ (MI:0019), since it is already in the results of ‘pull down’ experimental method with 0.83 score (see the clean function in Figure 5). The terms, which already have a substring with higher score in the list, are also cleaned from the list (see the filter function in Figure 6). The remaining terms in the list of each experimental method are included to its initial query as expansion terms together with their cosine similarity scores. The cosine similarity scores of the initial query terms are set as 1.0. This procedure is summarized in Figure 7.

As an example consider the ‘two hybrid’ experimental method. The initial query terms that have word vectors for this method are ‘2-hybrid’, ‘two-hybrid’ and ‘y2h’. The term ‘yeast two-hybrid assays’ is among the most similar 100 terms for each of these three initial query terms, where the corresponding cosine similarity scores are 0.773, 0.838 and 0.826, respectively. Therefore, the term is included into the list of the ‘two hybrid’ method with the cosine similarity score 0.838. The other two occurrences of the term are eliminated. The term ‘gst pull down’ is eliminated while cleaning the list of the ‘two hybrid’ method, since it contains the term ‘pull down’ which is a name of the pull down experimental method (MI:0096). Likewise, the term ‘bifc’ is not included into the list, since it is listed as a synonym of the experimental method ‘bimolecular fluorescence complementation’ (MI:0809) in the PSI-MI ontology. Moreover, the term ‘tap-tag’ with cosine score 0.739 is eliminated, since it is listed in the list of ‘tandem affinity purification’ (MI:0676) experimental method with cosine score 0.804. The term ‘yeast two-hybrid screening’ with cosine score 0.853 is eliminated since ‘two-hybrid screening’ is a substring of ‘yeast two-hybrid screening’, and its score is higher (0.860). The final expanded query for the ‘two hybrid’ experimental method is shown in Table 6. As a result, for each experimental method, an expanded query with a different size is obtained.

**Table 6.** The expanded query terms of ‘two hybrid’ (MI:0018) are shown in bold

Terms	Scores
**two hybrid**	**1.0**
**two-hybrid**	**1.0**
**yeast two hybrid**	**1.0**
**2 hybrid**	**1.0**
**2-hybrid**	**1.0**
**y2h**	**1.0**
**classical two hybrid**	**1.0**
**gal4 transcription regeneration**	**1.0**
*yeast two-hybrid*	*0.91416*
*two-hybrid system*	*0.874584*
*y2h system*	*0.869421*
*two-hybrid experiments*	*0.865643*
**yeast-two-hybrid**	**0.865173**
*two-hybrid analysis*	*0.851562*
*y2h assay*	*0.845296*
**yeast-two hybrid**	**0.844966**
*two-hybrid assays*	*0.844023*
*y2h screens*	*0.843109*
*two-hybrid assay*	*0.842222*
*y2h assays*	*0.84187*
*yeast two-hybrid assays*	*0.837754*
*yeast two-hybrid y2h*	*0.832441*
*y2h experiments*	*0.830541*
*yeast two-hybrid system*	*0.826506*
*two-hybrid screening*	*0.825039*
*yeast two-hybrid assay*	*0.824025*
**yeast-2-hybrid**	**0.823851**
*yeast 2-hybrid*	*0.820577*
*y2h screen*	*0.81859*
*two-hybrid screens*	*0.810895*
*y2h screening*	*0.807603*
*y2h interactions*	*0.806635*
*yeast two-hybrid screens*	*0.794152*
*two-hybrid interaction*	*0.791841*
*y2h interaction*	*0.787684*
**ap-ms**	**0.78406**
*two hybrid y2h*	*0.780946*
*high-throughput yeast two-hybrid*	*0.778971*
**y2h-based**	**0.775805**
*two-hybrid screen*	*0.769459*
*yeast-two-hybrid y2h*	*0.766024*
*large-scale yeast two-hybrid*	*0.76319*
*yeast two-hybrid screening*	*0.758309*
**PPI**	**0.756688**
**split-ubiquitin**	**0.7553**
*yeast two-hybrid y2h assays*	*0.75367*
*yeast two-hybrid y2h screens*	*0.749456*
**bait-prey**	**0.748784**
*yeast 2-hybrid assays*	*0.745503*
*yeast two-hybrid y2h assay*	*0.742103*
*large-scale y2h*	*0.740383*
*yeast-two-hybrid experiments*	*0.739971*
*two-hybrid interactions*	*0.739497*
**interactors**	**0.737921**
*yeast two-hybrid screenings*	*0.737534*
**interacting proteins**	**0.73329**
*yeast two-hybrid screen*	*0.730519*
**tap-ms**	**0.728466**
**literature-curated interactions**	**0.725529**
**cty10-5d**	**0.725047**
**yll049wp**	**0.715317**
*mammalian two-hybrid*	*0.713345*
*y2h bait*	*0.711965*
**lexa-based**	**0.710877**
**ap/ms**	**0.708912**
**lexa fusions**	**0.708365**
**bait prey**	**0.707578**
*yeast-two-hybrid assay*	*0.707011*
**interaction partners**	**0.706699**
*yeast-two-hybrid system*	*0.706006*
**jnm1p**	**0.705212**
**bait**	**0.704263**
**y3h**	**0.703098**
*bait plasmid*	*0.700969*
*putative interactors*	*0.700491*
**matchmaker gold**	**0.698625**
**mating-based**	**0.696109**
*bait construct*	*0.695515*
**co-ap**	**0.695304**
**yeast co-transformation**	**0.695194**
**mbsus**	**0.694528**
**interacting partners**	**0.694077**
**protein-interaction**	**0.692748**
**PPIs**	**0.692531**
**yeast strain y190**	**0.692009**
**matchmaker gal4**	**0.691998**
**ht-y2h**	**0.6908**
**domain gal4-ad**	**0.689826**
*yeast-two-hybrid assays*	*0.689708*
**bait/prey**	**0.689438**
*large-scale ap-ms*	*0.68708*
*y2h library screening*	*0.686014*
**biogrid**	**0.685742**
**prey-prey**	**0.685525**
*two-hybrid library*	*0.683535*
*high-throughput y2h*	*0.679981*
*yeast-two-hybrid screen*	*0.678596*
**yth assays**	**0.678315**
*y2h screenings*	*0.678036*
**high-confidence interactions**	**0.677115**
*yeast-two hybrid assays*	*0.655188*
*glutathione s-transferase gst pulldown*	*0.654585*
*gst-pulldown assay*	*0.646435*
**yeast three-hybrid assay**	**0.636332**
*gst-pulldown experiments*	*0.635499*
*yeast two-hybrid co-immunoprecipitation*	*0.635368*
*vitro pull-down experiments*	*0.634054*
*gst-pull-down assay*	*0.63086*
*gst-pull-down*	*0.628426*
**unc-89 ig1**	**0.62706**
*bait constructs*	*0.626819*
*gst-pull-down assays*	*0.626504*
**l40 yeast**	**0.625285**
**yeast strain mav203**	**0.624612**
**glutathione s-transferase gst tagged**	**0.624112**
*gst pulldowns*	*0.623283*
*y3h assays*	*0.622409*
**lexa-esc4**	**0.618557**
**eif2b***ϵ*	**0.618421**

The italic terms are eliminated from the word2vec results in the cleaning operation as explained in Figure 5. The terms with score 1.0 are the initial query items (name or synonyms). The terms which already contain (after splitting with space) any name or synonym are also eliminated, so italicized.

The generated expanded queries are used to identify the passages describing experimental methods in full text articles. Given a full text article in BioC format, the sentences in the paragraphs are matched against the expanded queries of each experimental method. The threshold for selecting a sentence as relevant to an experimental method is set to a certain value, e.g. 0.9 (for the word embeddings based approach) and 1.0 (for the baseline and tf.rf based approaches). If the query score for a sentence is greater than or equal to that threshold, the sentence is annotated with the experimental method for which it scored highest. The previous and next sentences of the selected sentence are also processed to check whether they are relevant to the same experimental method or not. If the previous and next sentences of the annotated sentence obtain the highest score for the same experimental method and if this score is greater than or equal to a certain value, e.g. 0.5 (for the baseline and tf.rf based approaches) and 0.65 (for the word embeddings based approach), they are annotated with the same experimental method. All the successive sentences with the same annotation are concatenated under one annotation tag. As a result, sentences or groups of sentences (passages) in paragraphs are annotated for experimental methods.

## Evaluation

### Jaccard index for passage similarity

The Jaccard index (44) is a statistic to measure similarity of given finite sets by taking the ratio of the size of the intersection and size of the union of the given sets as shown in Equation (2). In contrast, the Jaccard distance, which measures dissimilarity of given sets, is obtained by subtracting the Jaccard coefficient from 1 as shown in Equation (3).

(2) $$J(A,B)=\frac{\left|A\cap B\right|}{\left|A\cup B\right|}=\frac{\left|A\cap B\right|}{\left|A|+|B|-|A\cap B\right|}$$

(3) $$d_{J}(A,B)=1-J(A,B)$$

In our case, we use the Jaccard index to calculate the similarity of passages. Each passage can be thought as an ordered sequence of characters (string). When the Jaccard similarity of two passages is measured, the character length of each passage (|A| and |B|) and the character length of the intersection of the passages (|A∩B|) are calculated. The intersection of two passages can be in one of the following cases; (i) one passage can cover the other one, so the shorter passage is the intersection, (ii) the two passages can be completely the same, so any of them is the intersection, (iii) if case (i) or case (ii) are not satisfied, the longest common substring of the two passages is extracted as the intersection. After the length of each passage and the length of their intersection are calculated, we can measure the Jaccard similarity of the two passages according to Equation (2). The Jaccard index is between [0, 1]. The Jaccard indexes of two exactly the same passages are 1.0, whereas it is 0.0 for completely different passages.

### Evaluation measures

The performance of the system is evaluated by comparing the output articles of the system against the manually annotated versions. Each output article of the system is compared with its manually annotated version at paragraph level. Two annotations should have the same experimental method id and (fully or partially) common text to be evaluated as matched annotations. If this is not the case, the annotations are assessed as non-matched. The possible matched and non-matched cases are listed below;

- If a paragraph contains annotated passages in the manually annotated article, but the corresponding paragraph in the system output article does not have any annotated passages, this corresponds to the case of false negative (FN). The Jaccard distance for each annotated passage in the manually annotated paragraph is 1.0. The sum of those Jaccard distances is added to the total FN score of the evaluation.
- If a paragraph does not have any annotated passages in the manually annotated article, but the corresponding paragraph in the system output article has annotated passages, this corresponds to the case of FP. The Jaccard distance for each annotated passage in the system output paragraph is 1.0. The sum of those Jaccard distances is added to the total FP score of the evaluation.
- If a paragraph has annotated passages both in the manually annotated article and the system output article, but some of these annotations are non-matched (i.e. either the experimental method ID does not match or the annotated passages do not have any overlapping text), then these correspond to the cases of FN and/or FP. For each non-matched passage annotation in the manually annotated article, the FN score is updated as defined in case (i) earlier. At the same time, for each non-matched passage annotation in the system output article, the FP score is updated as defined in case (ii) earlier.
- If a paragraph has matched annotated passages both in the manually annotated article and the system output article, this corresponds to the case of true positive (TP). In this case, the Jaccard indexes of those passage pairs are added to the total TP score of the evaluation. In case of exact match, the Jaccard index for a passage pair is 1.0. However, if a passage pair matches partially in terms of common text, after its Jaccard index is added to the TP, we calculate the ‘Partial Jaccard Distance’ (our adaptation of Jaccard distance to this problem) for the unmatched parts of the passages as shown in Equations (6) and (7). The text of the manually annotated passage and the text of the system output passage are represented with M and S, respectively. The unmatched text portion can be part of either the manually annotated passage or system annotated passage. If it is part of the manually annotated passage, the partial Jaccard distance of the manually annotated passage (M) (Equation 6) is added to the FN score in the evaluation. Otherwise, if it is part of the system annotated passage, the partial Jaccard distance of the system annotated passage (S) (Equation 7) is added to the FP score. The partial Jaccard distance of a target passage from another passage is calculated by subtracting the Jaccard index of the passage pair from the normalized length of the target passage. The normalized length of the target passage of a passage pair can be calculated by taking the ratio of the character length of the target passage and the character length of the union of the passage pair as shown in Equations (4) and (5).
  (4) $$N_{M}(M,S)=\frac{\left|M\right|}{\left|M\cup S\right|}$$
  (5) $$N_{S}(M,S)=\frac{\left|S\right|}{\left|M\cup S\right|}$$
  (6) $${\text{pd}}_{\text{JM}}(M,S)=N_{M}(M,S)-J(M,S)$$
  (7) $$pd_{\text{JS}}(M,S)=N_{S}(M,S)-J(M,S)$$

**Figure 8.**
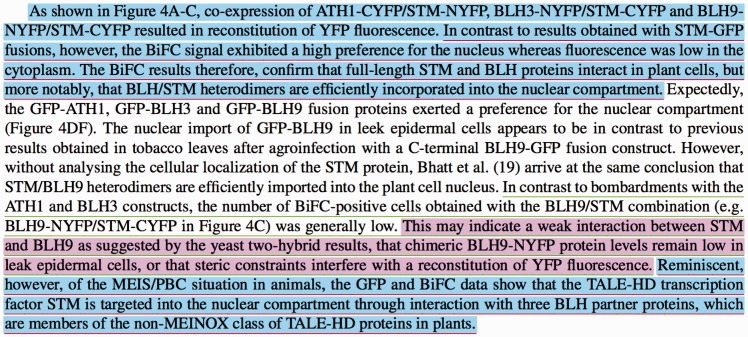
An example paragraph which shows our evaluation logic over three sample manual and system annotations. The manually annotated passages are underlined with red and green for ‘bimolecular fluorescence complementation’ (MI:0809) and ‘two hybrid’ (MI:0018) experimental methods, respectively. The annotated passages by the system are colored with blue and purple for ‘bimolecular fluorescence complementation’ (MI:0809) and ‘two hybrid’ (MI:0018) experimental methods, respectively.

The following example, which is shown in Figure 8, covers the different cases mentioned above for the evaluation logic of the annotated passages. The paragraph in Figure 8 is taken from (45). This article is also in our published manually annotated data set (https://github.com/ferhtaydn/biocemid/blob/odj/files/published_dataset/1651 3846 . xml).

In the first annotation, the system identified the manually annotated passage fully. However, it incorrectly included the first sentence to the passage as well, which resulted in a FP text portion. The manual annotation text length is 371 characters, whereas the system annotation text length is 523 characters. The union length is 523 and matching (common) text length is 371. Since the Jaccard index is (371/523)=0.709, TP is increased with 0.709. FN is not changed (the manual annotation is fully covered). The partial Jaccard distance is calculated as ((523−371)/523)=0.291 and FP is increased with 0.291. In the second annotation, the system did not annotate any additional incorrect sentences. However, it was not able to identify the manually annotated passage fully, but identified only a portion of it. The manual annotation text length is 452 characters and system annotation text length is 258 characters. The union length is 452 and matching text length is 258 characters. Since the Jaccard index is (258/452)=0.571, TP is increased with 0.571. FP is not changed. The partial Jaccard distance is calculated as ((452−258)/452)=0.429 and FN is increased with 0429. The last annotation is an exact match. The manual and system annotations are the same. Thus, the Jaccard index is 1.0 and TP is increased with 1.0. FN and FP are not changed.

After the calculation of the TP, FP and FN scores for all passages in the articles, Recall, Precision and *F*-measure are calculated as shown in Equations (8)–(10), respectively.

(8) $$\text{Recall}=\frac{\text{TP}}{\text{TP}+\text{FN}}$$

(9) $$\text{Precision}=\frac{\text{TP}}{\text{TP}+\text{FP}}$$

(10) $$F-\text{measure}=\frac{2*\text{Precision}*\text{Recall}}{\text{Precision}+\text{Recall}}$$

## Results

The developed methods (‘baseline’, ‘tf.rf’ and ‘word.embeddings’) are evaluated under different configurations on the test set, which comprises 17 full text articles, and the performances of the methods are compared with each other as shown in Table 7. The baseline approach does not need a training or validation set, since the existence of a name or a synonym of an experimental method determines the result of the annotation for that sentence. There is no a training or parameter tuning phase. On the other hand, the tf.rf-based approach is supervised and needs a training set to extract the Tier 1 and Tier 2 terms to expand the initial queries. The training set of 13 articles is used for that purpose in the tf.rf method. Like in the baseline, the threshold value, which determines whether a sentence should be annotated with an experimental method, is determined heuristically (without tuning) as explained in the ‘Baseline for query matching’ and ‘tf.rf-based query generation’ sections. Since the ‘word.embeddings’ based method is an unsupervised approach, there is no need for a training set, so the 13 articles are used as validation set to determine the threshold values for the main target sentence and the previous and next sentences around the target sentence for annotation.

**Table 7.** Performances of the methods on the test set

	Precision	Recall	*F*-measure
baseline	0.424	0.418	0.421
baseline.genia.ino	0.484	0.413	0.446
tf.rf.f7s7	0.120	0.508	0.194
tf.rf.f7s7.genia.ino	0.133	0.502	0.211
tf.rf.f10s10	0.068	0.512	0.119
tf.rf.f10s10.genia.ino	0.074	0.507	0.129
tf.rf.manual	0.315	0.508	0.389
tf.rf.manual.genia.ino	0.357	0.503	0.418
word2vec	0.321	0.618	0.422
word2vec.genia.ino	0.362	0.606	0.453

In the ‘tf.rf’-based approach, we experimented with three different configurations as explained in the ‘tf.rf-based query generation’ section. The manual selection of the tf.rf terms is labeled as ‘tf.rf.manual’ and the automatic selections of the tf.rf terms are labeled as ‘tf.rf.f7s7’ and ‘tf.rf.f10s10’ in the results table. ‘tf.rf.f7s7’ corresponds to the configuration when the first 7 and the second 7 terms are included in the Tier 1 and Tier 2 lists, respectively. Similarly, ‘tf.rf.f10s10’ corresponds to the configuration when the first 10 and the second 10 terms are included in the Tier 1 and Tier 2 lists, respectively. The results in Table 7 show that all three tf.rf configurations obtain similar recall levels, which are higher than the recall of the baseline. The precision values of the tf.rf configurations with automatically selected terms are much lower than the tf.rf configuration with manually selected terms, all of which are lower than the precision of the baseline. The results also show that including more automatically selected terms to the tr.rf approach leads to only a slight increase in recall, but results in drastic decrease in precision and *F*-measure.

In the ‘word.embeddings’ (word2vec) based approach; the threshold for detecting the main target sentences is set as 0.9 and the threshold for detecting the before and after sentences of the main target sentences is set as 0.65. These thresholds were determined after running the system, on the validation set of 13 articles, with a range of different threshold values to determine the best setting in terms of highest *F*-measure performance. While improving the recall and *F*-measure scores (i.e. improvement in FN and TP scores), we try to improve (i.e. decrease) the FP score by applying an extra constraint in the annotation decision step, which mandates a passage to contain at least one protein and one interaction keyword. Passages which explain the details of how an experiment was conducted (i.e. the experiment layout) without giving any information/explanation or evidence about the interacting proteins are prevented to be annotated by this constraint. Likewise, passages that only include some experimental method related keywords, but do not give any detail about the proteins or the interaction are eliminated. Protein name detection in the passages is done with the Genia Tagger (http://www.nactem.ac.uk/tsujii/GENIA/tagger/) (GENIA Project: Mining literature for knowledge in molecular biology, Version: 3.0.2, RRID:SCR_007990) (46). To detect the interaction keyword(s) in the passages, Interaction Network Ontology (INO) (http://www.ino-ontology.org/, http://bioportal.bioontology.org/ontologies/INO) (Interaction Network Ontology, Version: 1.0.95, RRID: SCR_010347) (47, 48) is used. The terms under the ‘INO_0000006’ annotation (http://purl.obolibrary.org/obo/INO_0000006), defining the literature mining keywords, are extracted and the protein related interaction keywords are filtered manually (https://github.com/ferhtaydn/biocemid/blob/odj/files/ino/literature_mining_keywords_related_to_protei ns . txt). As illustrated in Table 7, this constraint results in considerable improvement in precision and *F*-measure for all methods (represented with the ‘genia.ino’ extension) with a relatively smaller decrease in recall.

The goal of the BioC Task was to develop a text-mining system to assist biocurators. It has been shown that high recall is more desirable than high precision for a system aiming to assist biocurators in exhaustive curation (14). It is relatively easier for a biocurator to filter the FPs retrieved by the system compared with manually finding the FNs missed by the system. Therefore, we aimed at increasing the recall of the baseline method by query expansion without sacrificing much from precision. To the best of our knowledge, there does not exist a study on identifying passages describing PPI detection methods. So, there are no previously reported state of the art results/scores for this task. Therefore, we compare the proposed approaches with the performance of the baseline approach. As shown in the results table, the word embeddings based methods and the tf.rf based methods achieve higher recall scores than the baseline method at the expense of lower precision. In addition to higher recall, the word embeddings based approach achieves slightly higher *F*-measure than the baseline. In addition, it outperforms the tf.rf based methods in terms of precision, recall, and F-measure, which shows that it is a promising approach for detecting passages with experimental methods.

## Discussion

For a query matching based information retrieval system, it is crucial that queries are expanded with highly relevant terms to increase the overall performance of the system. The amount of data used to learn the relevant terms for query expansion in general affects the quality of the final expanded query. Supervised learning techniques need labeled data. Manual data labeling is a laborious and time consuming process. On the other hand, unsupervised learning techniques use unlabeled data. The amount of published articles in the biomedical domain constitutes a large unlabeled data set that can be used for unsupervised learning. Therefore, compared with supervised techniques, unsupervised techniques can help to scan a larger set of articles in the literature for more experimental methods to extract valuable information such as repeating patterns, sentence rules, experiment specific terms, proteins etc. in the experimental method related passages. Nevertheless, if an experimental method is not mentioned enough in the literature, the effort for extracting information related to that experimental method by unsupervised techniques would not give encouraging results. For example, running ‘word2vec’ on 691,558 articles (2,241,223,681 words) produced word embeddings for the names of only 58 out of 103 experimental methods (The list of experimental methods which have word embeddings in this study is available at https://github.com/ferhtaydn/biocemid/tree/odj/files/oa_word 2vecs_pure_baseline). For infrequently used (uncommon) experimental methods, manually curated rules or queries created by domain experts can be used to enhance the retrieval results by expanding such rare experimental methods’ queries with high quality terms.

There are some hard cases that need to be considered for improving the results. Experimental methods which are siblings or close to each other in the PSI-MI ontology have very similar names or synonyms, definitions and experimental details. For example, ‘anti bait coimmun opreci pitation’ (MI:0006) and ‘anti tag coimmunoprecipitation’ (MI:0007) are siblings, and ‘coimmunoprecipitation’ (MI:0019) is their parent. After the queries are expanded with unsupervised techniques like the word embeddings based approach, specific patterns or rules may be defined to distinguish such experimental methods from each other. The word embeddings based approach cannot expand the queries of ‘anti bait coimmunoprecipitation’ and ‘anti tag coimmunoprecipitation’ methods, since their names are not used directly as a method name in the text. Therefore, the system annotates a passage with ‘coimmunopr ecipi tation’, even if it is related to ‘anti bait coimmunoprecipi tation’ or ‘anti tag coimmunoprecipitation’. When labeling with the parent experimental method is considered correct, i.e. when the annotations, which have 0006 and 0007 values in the ‘PSIMI’ infon, are changed to 0019 in the manually annotated articles, the scores of ‘word2vec.genia.ino’ configuration increase as shown in Table 8 (‘word2vec .genia. ino.coip’). Moreover, when the system is evaluated in experimental method ID agnostic way (which means, the ID of the experimental method (i.e. the PSIMI infon) is not considered as long as the passage retrieved describes an experimental method), the scores increase considerably as shown in Table 8 (‘word2vec.genia.ino.psimi’).

**Table 8.** Additional results on the test set

	Precision	Recall	*F*-measure
**word2vec.genia.ino**	0.362	0.606	0.453
**word2vec.genia.ino.coip**	0.390	0.645	0.486
**word2vec.genia.ino.psimi**	0.439	0.751	0.554

The system evaluation when the ‘anti bait coimmunoprecipitation’ and ‘anti tag coimmunoprecipitation’ methods are regarded as ‘coimmunoprecipitation’ is shown with ‘word2vec.genia.ino.coip’. The system evaluation without the requirement of experimental method ID matching is shown with ‘word2vec.genia.ino.psimi’.

Ambiguous terms also require special focus. For example, the term ‘cross-linking’, which is a name of an experimental method for detecting physical PPIs (MI:0030), is also frequently used in articles to identify interactions between genes. As future work, we plan to investigate these edge cases and improve the performance of our system by better discriminating ambiguous terms and closely related experimental methods.

Another source of error is the passages that describe experimental layout (i.e. describe how an experiment was performed) without providing explicit evidence for PPI. In our data set, we did not manually annotate such passages. However, since they may contain experimental method names or related terms, our system can annotate such parts in the articles and this situation causes to an increase in FP rates. The performance of the system can be improved by defining the layout patterns or adding constraints into the algorithm to filter such cases.

## Conclusion

In this work, we defined the problem of extracting passages with experimental interaction detection methods used to determine physical interactions between proteins from full text articles in the biomedical domain as an information retrieval search task. In order to extract passages describing experimental methods as evidence for physical PPIs, an initial query for each experimental method is prepared by utilizing the names and synonyms of that experimental method from the PSI-MI ontology. The baseline approach is based on matching those initial queries to the sentences in the paragraphs of articles. To improve the performance of a query matching based approach, the existing queries can be expanded with more relevant terms. Therefore, we proposed two new approaches based on the baseline approach. The first method is supervised and based on expanding the initial queries with the terms determined with the tf.rf metric on manually annotated passages. Our second approach is unsupervised and based on expanding the queries with terms determined using the word emmbeddings of the terms of the initial queries. In addition, we annotated and made publicly available a data set of 30 full text articles labeled for passages describing experimental methods used to detect physical PPI.

We applied the tf.rf term weighting metric to passage level classification (instead of its original application to the article level text classification problem). We started from the idea that determining important and discriminating terms for each experimental method, by assigning appropriate weights, should help the problem of extracting passages and classifying them to the experimental methods that they describe. The tf.rf based methods achieve higher recall scores than the baseline method at the expense of lower precision and *F*-measure. We also applied distributional semantic models, by using the word2vec tool and generating word embeddings of terms specific to each experimental method defined in the ontology to expand the queries, for the passage extraction problem in the biomedical domain. The word embeddings based approach achieves considerably higher recall compared with the baseline and slightly better *F*-measure. In addition, the word embeddings based approach outperforms the tf.rf based approach in terms of precision, recall and *F*-measure, which shows that utilizing the huge and growing biomedical literature within an unsupervised learning setting is an effective approach. We also showed that empowering the proposed approaches with extra constraints or features like terms from domain specific ontologies (i.e. interaction keywords from INO) and specific named entities (i.e. protein names, determined with the GENIA Tagger, in the sentences) leads to improved performance.

The most challenging part of this study was gathering enough labeled data. Manually annotating passages for multi-class of experimental methods in full text domain specific articles is a difficult and time consuming task. The labeled data are needed for training, validation and as test (gold standard) sets to develop, validate and test the proposed approaches. The lack of enough labeled data for supervised approaches (as training set), like in our tf.rf based approach, limits the learning ability of the algorithms.

An automatic passage (context) extraction system could be a valuable tool for the biomedical domain. It can help to reduce the time consumed on curation for biocurators. In addition, it can help scientists reach relevant information in a structured format by facilitating the expansion of existing databases. We plan to enlarge the target experimental method list and manually annotated data set while trying to solve the edge cases defined in the ‘Discussion’ section.

## References

[1] Phizicky E.M. Fields S. (1995) Protein-protein interactions: methods for detection and analysis. Microbiol. Rev., 59, 94–123. 7708014 10.1128/mr.59.1.94-123.1995 PMC239356

[2] Chatr-Aryamontri A. Breitkreutz B.J. Oughtred R. (2015) The biogrid interaction database: 2015 update. Nucleic Acids Res., 43, D470–D478. 25428363 10.1093/nar/gku1204 PMC4383984

[3] Hermjakob H. Montecchi-Palazzi L. Lewington C. (2004) Intact: an open source molecular interaction database. Nucleic Acids Res., 32, D452–D455. 14681455 10.1093/nar/gkh052 PMC308786

[4] Xenarios I. Salwinski L. Duan X.J. (2002) Dip, the database of interacting proteins: a research tool for studying cellular networks of protein interactions. Nucleic Acids Res., 30, 303–305. 11752321 10.1093/nar/30.1.303 PMC99070

[5] Chatr-Aryamontri A. Ceol A. Palazzi L.M. (2007) Mint: the molecular interaction database. Nucleic Acids Res., 35, D572–D574. 17135203 10.1093/nar/gkl950 PMC1751541

[6] Bader G.D. Betel D. Hogue C.W. (2003) Bind: the biomolecular interaction network database. Nucleic Acids Res., 31, 248–250. 12519993 10.1093/nar/gkg056 PMC165503

[7] Baumgartner W.A. Cohen K.B. Fox L.M. (2007) Manual curation is not sufficient for annotation of genomic databases. Bioinformatics, 23, i41–i48. 17646325 10.1093/bioinformatics/btm229 PMC2516305

[8] Arighi C.N. Lu Z. Krallinger M. (2011) Overview of the biocreative iii workshop. BMC Bioinformatics, 12, S1. 10.1186/1471-2105-12-S8-S1 PMC3269932 22151647

[9] Hirschman L. Yeh A. Blaschke C. Valencia A. (2005) Overview of biocreative: critical assessment of information extraction for biology. BMC Bioinformatics, 6, S1. 10.1186/1471-2105-6-S1-S1 PMC1869002 15960821

[10] Krallinger M. Morgan A. Smith L. (2008) Evaluation of text-mining systems for biology: overview of the second biocreative community challenge. Genome Biol., 9, S1. 10.1186/gb-2008-9-s2-s1 PMC2559980 18834487

[11] Kim J.D. Ohta T. Pyysalo S. (2009) Overview of bionlp’09 shared task on event extraction. In: *Proceedings of the Workshop on Current Trends in Biomedical Natural Language Processing: Shared Task. BioNLP ’09, Association for Computational Linguistics*, Stroudsburg, PA, USA, pp. 1–9.

[12] Kim J.D. Pyysalo S. Ohta T. (2011) Overview of bionlp shared task 2011. In: *Proceedings of the BioNLP Shared Task 2011 Workshop. Association for Computational Linguistics*, Portland, OR, USA, pp. 1–6.

[13] Nédellec C. Bossy R. Kim J.D. (2013) Overview of bionlp shared task 2013. In: Proceedings of the BioNLP Shared Task 2013 Workshop, pp. 1–7.

[14] Krallinger M. Leitner F. Rodriguez-Penagos C. (2008) Overview of the protein-protein interaction annotation extraction task of biocreative ii. Genome Biol., 9, S4. 10.1186/gb-2008-9-s2-s4 PMC2559988 18834495

[15] Tikk D. Thomas P. Palaga P. (2010) A comprehensive benchmark of kernel methods to extract protein–protein interactions from literature. PLoS Comput. Biol., 6, e1000837. 20617200 10.1371/journal.pcbi.1000837 PMC2895635

[16] Krallinger M. (2010) Importance of negations and experimental qualifiers in biomedical literature. In: Proceedings of the Workshop on Negation and Speculation in Natural Language Processing. Association for Computational Linguistics, pp. 46–49.

[17] Rinaldi F. Kappeler T. Kaljurand K. (2008) Ontogene in biocreative ii. Genome Biol., 9, S13. 18834491 10.1186/gb-2008-9-s2-s13 PMC2559984

[18] Ehrler F. Gobeill J. Tbahriti I. Ruch P. (2007) Geneteam site report for biocreative ii: Customizing a simple toolkit for text mining in molecular biology. In: Proceedings of the Second BioCreative Challenge Evaluation Workshop: Madrid, Spain, pp. 199–207.

[19] Krallinger M. Vazquez M. Leitner F. (2011) The protein-protein interaction tasks of biocreative iii: classification/ranking of articles and linking bio-ontology concepts to full text. BMC Bioinformatics, 12, S3. 10.1186/1471-2105-12-S8-S3 PMC3269938 22151929

[20] Hermjakob H. Montecchi-Palazzi L. Bader G. (2004) The hupo psi’s molecular interaction format a community standard for the representation of protein interaction data. Nat. Biotechnol., 22, 177–183. 14755292 10.1038/nbt926

[21] Kerrien S. Orchard S. Montecchi-Palazzi L. (2007) Broadening the horizon–level 2.5 of the hupo-psi format for molecular interactions. BMC Biol., 5, 44. 17925023 10.1186/1741-7007-5-44 PMC2189715

[22] Kappeler T. Clematide S. Kaljurand K. (2008) Towards automatic detection of experimental methods from biomedical literature. In: Third International Symposium on Semantic Mining in Biomedicine (SMBM), Turku Centre for Computer Science (TUCS).

[23] Lourenço A. Conover M. Wong A. (2011) A linear classifier based on entity recognition tools and a statistical approach to method extraction in the protein-protein interaction literature. BMC Bioinformatics, 12, 1. 22151823 10.1186/1471-2105-12-S8-S12 PMC3269935

[24] Jhamb D. Krishnan A. Palakal M. (2014) Identification of protein interaction methods from biomedical literature. In: Computational Advances in Bio and Medical Sciences (ICCABS), 2014 IEEE 4th International Conference on, Miami Beach, FL, USA, pp. 1–6.

[25] Danger R. Pla F. Molina A. Rosso P. (2014) Towards a protein–protein interaction information extraction system: Recognizing named entities. Knowledge-Based Syst., 57, 104–118.

[26] Matos S. Campos D. Oliveira J.L. (2010) Vector-space models and terminologies in gene normalization and document classification. In: Proceedings of the BioCreative III Workshop, Citeseer, pp. 119–124.

[27] Schneider G. Clematide S. Rinaldi F. (2011) Detection of interaction articles and experimental methods in biomedical literature. BMC Bioinformatics, 12, 1. 22151872 10.1186/1471-2105-12-S8-S13 PMC3269936

[28] Agarwal S. Liu F. Yu H. (2011) Simple and efficient machine learning frameworks for identifying protein-protein interaction relevant articles and experimental methods used to study the interactions. BMC Bioinformatics, 12, S10. 10.1186/1471-2105-12-S8-S10 PMC3269933 22151701

[29] Wang X. Rak R. Restificar A. (2011) Detecting experimental techniques and selecting relevant documents for protein-protein interactions from biomedical literature. BMC Bioinformatics, 12, S11. 10.1186/1471-2105-12-S8-S11 PMC3269934 22151769

[30] Soulat D. Bürckstümmer T. Westermayer S. (2008) The dead-box helicase ddx3x is a critical component of the tank-binding kinase 1-dependent innate immune response. EMBO J., 27, 2135–2146. 18583960 10.1038/emboj.2008.126 PMC2453059

[31] Aydın F. Hüsünbeyi Z.M. Ozgür A. (2015) Retrieving passages describing experimental methods using ontology and term relevance based query matching. In: Proceedings of the Fifth BioCreative Challenge Evaluation Workshop, Sevilla, Spain, pp. 42–50.

[32] Comeau D.C. Doğan R.I. Ciccarese P. (2013) Bioc: a minimalist approach to interoperability for biomedical text processing. Database, 2013, bat064. 24048470 10.1093/database/bat064 PMC3889917

[33] Kim S. Dogan R.I. Chatr-Aryamontri A. (2015) Overview of biocreative v bioc track. In: Proceedings of the Fifth BioCreative Challenge Evaluation Workshop, Sevilla, Spain, pp. 1–9.

[34] Kim S. Doğan R.I. Chatr-Aryamontri A. (2016) Biocreative v bioc track overview: collaborative biocurator assistant task for biogrid. Database, 2016, baw121. 27589962 10.1093/database/baw121 PMC5009341

[35] Mikolov T. Chen K. Corrado G. Dean J. (2013) Efficient estimation of word representations in vector space. arXiv Preprint arXiv:1301.3781.

[36] Mikolov T. Yih W. Zweig G. (2013) Linguistic regularities in continuous space word representations. In: hlt-Naacl, Association for Computational Linguistics, pp. 746–751.

[37] Maloney C. Sequeira E. Kelly C. Pubmed central (2013) In: The NCBI Handbook [Internet]. 2nd edition. Bethesda (MD): National Center for Biotechnology Information (US); 2013.

[38] Hripcsak G. Rothschild A.S. (2005) Agreement, the F-measure, and reliability in information retrieval. J. Am. Med. Informatics Assoc., 12, 296–298. 10.1197/jamia.M1733 PMC1090460 15684123

[39] Manning C.D. Surdeanu M. Bauer J. (2014) The Stanford CoreNLP natural language processing toolkit. In: Association for Computational Linguistics (ACL) System Demon strations. pp. 55–60, http://www.aclweb.org/anthology/P/P14/P14-5010.

[40] Lan M. Tan C.L. Su J. Lu Y. (2009) Supervised and traditional term weighting methods for automatic text categorization. Pattern Analysis and Machine Intelligence, IEEE Transactions on, vol. 31, pp. 721–735. 10.1109/TPAMI.2008.110 19229086

[41] Erk K. (2012) Vector space models of word meaning and phrase meaning: a survey. Lang. Linguist. Compass, 6, 635–653.

[42] Morin F. Bengio Y. (2005) Hierarchical probabilistic neural network language model In: Cowell R.G. Ghahramani Z. (eds.) Proceedings of the Tenth International Workshop on Artificial Intelligence and Statistics. Society for Artificial Intelligence and Statistics, pp. 246–252.

[43] Mnih A. Hinton G.E. (2009) A scalable hierarchical distributed language model. In: Advances in Neural Information Processing Systems, Curran Associates, Inc. pp. 1081–1088.

[44] Jaccard P. (1908) Nouvelles recherches sur la distribution florale. Bull. Socit Vaudoise Sci. Nat., 44, 223–270.

[45] Cole M. Nolte C. Werr W. (2006) Nuclear import of the transcription factor shoot meristemless depends on heterodimerization with blh proteins expressed in discrete sub-domains of the shoot apical meristem of arabidopsis thaliana. Nucleic Acids Res., 34, 1281–1292. 16513846 10.1093/nar/gkl016 PMC1388269

[46] Tsuruoka Y. Tateishi Y. Kim J.D. (2005) Developing a robust part-of-speech tagger for biomedical text. In: Panhellenic Conference on Informatics. Springer, Volos, Greece, pp. 382–392.

[47] Özgür A. Xiang Z. Radev D.R. He Y. (2011) Mining of vaccine-associated ifn-*γ* gene interaction networks using the vaccine ontology. J. Biomed. Seman., 2, 1. 10.1186/2041-1480-2-S2-S8 PMC3102897 21624163

[48] Hur J. Özgür A. Xiang Z. He Y. (2015) Development and application of an interaction network ontology for literature mining of vaccine-associated gene-gene interactions. J. Biomed. Seman., 6, 1. 10.1186/2041-1480-6-2 PMC4362819 25785184

